# New mechanism of plasmons specific for spin-polarized nanoparticles

**DOI:** 10.1038/s41598-019-38657-w

**Published:** 2019-02-14

**Authors:** Hari L. Bhatta, Ali E. Aliev, Vladimir P. Drachev

**Affiliations:** 1 Department of Physics and Advanced Materials and Mechanical Processing Institute, University of North Texas, Denton, TX 76203 USA; 2 A. G. MacDiarmid NanoTech Institute, University of Texas at Dallas, Richardson, TX 75083 USA; 3 Skolkovo Institute of Science and Technology, Moscow, 121205 Russia

## Abstract

Here it is experimentally shown that Co nanoparticles with a single-domain crystal structure support a plasmon resonance at approximately 280 nm with better quality than gold nanoparticle resonance in the visible. Magnetic nature of the nanoparticles suggests a new type of these plasmons. The exchange interaction of electrons splits the energy bands between spin-up electrons and spin-down electrons. It makes it possible for coexistence of two independent channels of conductivity as well as two independent plasmons in the same nanoparticle with very different electron relaxation. Indeed, the density of empty states in a partially populated d-band is high, resulting in a large relaxation rate of the spin-down conduction electrons and consequently in low quality of the plasmon resonance. In contrast, the majority electrons with a completely filled d-band do not provide final states for the scattering processes of the conduction spin-up electrons, therefore supporting a high quality plasmon resonance. The scattering without spin flip is required to keep these two plasmons independent.

## Introduction

The Mott model^1,2^ of conductivity in magnetic metals introduces three main points. *First*, the electrical conductivity in metals can be described in terms of two largely independent conducting channels, corresponding to the spin-up and spin-down electrons, which are distinguished according to the projection of their spins along the quantization axis. *Second*, the probability of spin-flip scattering processes in metals is normally small compared to the probability of the scattering processes in which the spin is conserved. This means that the spin-up and spin-down electrons do not mix over long distances and, therefore, the electrical conduction occurs in parallel for the two spin channels. *Third*, the scattering rates in ferromagnetic metals of the spin-up and spin-down electrons are quite different, whatever the nature of the scattering centers is. These two channels of conductivity with a distinct spin-dependent scattering is the primary origin of giant magnetoresistance^3^. Here, these ideas of two independent channels have been projected onto collective electron oscillations in spin-polarized nanoparticles. Understanding the effect of spin polarization on plasmon oscillations of the free electrons in nanoparticles is, essentially, unexplored and crucial in many envisioned applications at the cross road of magnetism and plasmonics. Particularly, it results in a new type of plasmon, specific for spin-polarized magnetic nanoparticles.

It is a common belief that the quality of the plasmon resonance of magnetic nanoparticles such as Co is quite low, which follows, in particular, from the experimental data for permittivity of bulk cobalt by Johnson and Christy (J&C)^4^. Here we show that for single-domain magnetic nanoparticles the usual approach, based on bulk permittivity, does not hold, though it did for perfectly for nonmagnetic (diamagnetic) nanoparticles. Indeed, our experiments prove that Co nanoparticles with a single-domain crystal structure support a sharp plasmon resonance at about 280 nm with the resonance quality comparable to gold nanoparticles. This type of plasmons is quite different from known plasmons in noble metals. Following Mott’s arguments there are two independent plasmons which co-exist in a spin-polarized metal nanoparticle. These two plasmons coexist in a particle at the same frequency and polarizations of excitation, but for electrons of opposite spin. The very different relaxation rates for two opposite spins make the spectral width of two plasmon resonances also very different, broad and narrow. Inter-nanoparticle interactions completely demolish plasmon quality resonance, which is the probable reason why it was not observed previously and why the results for bulk films^4^ cannot be used for single domain nanoparticles evaluations. It is known that the exchange interaction of electrons splits the energy bands between spin-up (majority) electrons and spin-down (minority) electrons. We suggest that a low quality of the plasmon resonance for spin-down electrons is due to the large relaxation rate of the conduction electrons caused by high density of empty states in a partially populated d-band. However, the majority electrons with a completely filled d-band does not affect the relaxation rate and plasmon resonance of the conduction spin-up electrons within magnetic nanoparticles. Note that the plasmon resonance of Co is in the deep ultraviolet spectral range, which is the range for bio-molecule resonances and therefore attractive for bio-medical applications as well as for its magnetic nature.

## Results

Cobalt nanoparticles were synthesized using a method similar to that of Sun and Murray^5^. The reduction of cobalt nanoparticles was conducted under inert atmosphere. At room temperature, 0.13 g (0.019 M) of anhydrous cobalt chloride, 0.3 mL (0.018 M) of oleic acid and 30 mL (1.87 M) of dioctyl ether were mixed together under purged nitrogen gas in the three- necked flask containing magnetic stir bar and heated to 100 °C. Then 1.5 mL (0.063 M) of trioctylphosphine, which was injected via syringe and the temperature raised to 205 °C. At this temperature, a strong reducing reagent, 1.5 mL (0.236 M) lithium triethylborohydride, was added in solution and the cobalt nanoparticles begin to grow immediately. The blue colour of the solution turns to black upon nucleation and growth of cobalt nanoparticles. The reaction was terminated by cooling the solution to room temperature and 20 mL (4.8 M) of anhydrous ethanol was added to precipitate the particles. The solution was aged overnight at room temperature in order to attach cobalt nanoparticles to the magnetic stir bar in the flask. The cobalt nanoparticles are removed from a magnetic stir bar and washed several times with ethanol by centrifugation. Finally, oleic acid coated cobalt nanoparticles were suspended in 8 mL of hexane.

To address the mechanism of new type of plasmons specific for magnetic nanoparticles our work involves the structural electron microscopy, SQUID magnetometry, dynamic light scattering (DLS), and spectroscopy of Co nanoparticles. The structural and magnetic characterizations prove the single-domain and superparamagnetic properties of nanoparticles required for spin dependent channels of plasmon oscillations. In the presence of sonication, the magnetic field-induced aggregation of nanoparticles in our experiments results in the suppression of the resonance quality.

**Figure 1:**
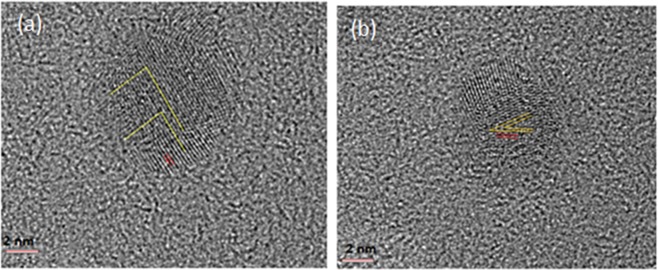
(**a**) High resolution TEM pattern of [100] HCP crystal structure of Co nanoparticle. (**b**) High resolution TEM pattern of [111] FCC crystal structure of Co nanoparticle.

An energy dispersive X-ray (EDX) spectrum analysis clearly shows the presence of cobalt peaks as presented in the Supporting Information (SI). High resolution TEM images were obtained with the FEI Tecnai G2 F20 S-Twin 200 keV field emission scanning transmission electron microscope (S/TEM). The high magnification TEM image of spherical cobalt nanoparticles shows that the size distribution of cobalt nanoparticles ranges from 6–12 nm with average particle diameter of 8.7 nm (See SI for more details on TEM characterization). High resolution TEM images show that our particles form both hcp and fcc crystal structure. Figure 1a shows TEM of [100] crystal structure of some cobalt nanoparticles have lattice spacing 2.102 Å and inter-planar angle 83.6° typical for hcp. Figure 1b shows high resolution TEM image of [111] crystal structure of other cobalt nanoparticles have lattice spacing 1.95 Å and inter-planar angle 25.63°, typical for fcc.

High quality plasmon resonance in absorption is proven below to be the property of isolated Co nanoparticles. Indeed, we observe a complete suppression of sharp plasmon resonance for aggregated Co nanoparticles, probably due to the interparticle interaction inducing a spin-flip electron scattering at the particle surface. This behavior is reversible, i.e., the sharp resonance is completely restored for separated nanoparticles after sonication, as shown below. Note that the absence of cobalt oxide shell, which could introduce an antiferromagnetic response, is controlled with the low temperature SQUID measurements (see SI Figs S1.8 and S1.9).

The ab-initio simulations of the relaxation constants performed for the giant magnetoresistance show a large difference for spin-up and spin-down electrons^6,7^. Susceptibility of Co nanoparticles of radius *a* can be expressed as a sum of two terms ${\chi }_{i}\{i=\,\uparrow \,,\downarrow \,\}$ coming from two independent groups of electrons. Thus, the total polarizability at frequency $\omega$ is given by:

1 $$\alpha (\omega )={r}^{3}({\chi }_{\uparrow }+{\chi }_{\downarrow })$$

Here we use the spectral representation of the Drude-Sommerfield model^8,9^.

2 $${\chi }_{i}=\frac{{\varepsilon }_{h}-{\varepsilon }_{mi}}{2{\varepsilon }_{h}+{\varepsilon }_{mi}}=\frac{1}{{X}_{i}+i{\delta }_{i}}$$

where ${\varepsilon }_{h},\,{\varepsilon }_{mi}$ permittivities of a host medium and metal, ${\varepsilon }_{mi}={\varepsilon }_{bi}-\frac{{\omega }_{p}^{2}}{\omega (\omega +i2{{\rm{\Gamma }}}_{i})}$, ${\varepsilon }_{bi}$ is a contribution of the interband transitions, ${{\rm{\Gamma }}}_{i}$ is the free electron relaxation rate, the plasma frequency ${\omega }_{p}={(n{e}^{2}/{\varepsilon }_{0}m)}^{1/2}$ is a function of the electron concentration $n$, other notations include electron charge and mass, and the permittivity of free space. Thus the spectral parameters ${X}_{i}=\frac{{\omega }_{sp}^{2}-{\omega }^{2}}{{\omega }_{sp}^{2}},{\delta }_{i}=\frac{\omega 2{{\rm{\Gamma }}}_{i}}{{\omega }_{sp}^{2}},{\rm{where}}\,{\omega }_{sp}^{2}=\frac{{\omega }_{p}^{2}}{{\varepsilon }_{bi}+2{\varepsilon }_{h}},$ and implying ${\varepsilon }_{h}\approx {\varepsilon }_{bi}$. Note that $2{{\rm{\Gamma }}}_{\uparrow }={\upsilon }_{F}/{\lambda }_{\uparrow }$ and $2{{\rm{\Gamma }}}_{\downarrow }={\upsilon }_{F}/{\lambda }_{\downarrow }$, where ab initio calculations for bulk Co give ${\lambda }_{\uparrow }=12\,\mathrm{nm},{\lambda }_{\uparrow }/{\lambda }_{\downarrow }\,{\rm{can}}\,{\rm{be}}\,\mathrm{10}-\mathrm{20}$^6^, and Fermi velocity ${\upsilon }_{F}=2.1\times {10}^{5}m/s$^7^. Extinction cross-section is kImα.

**Figure 2:**
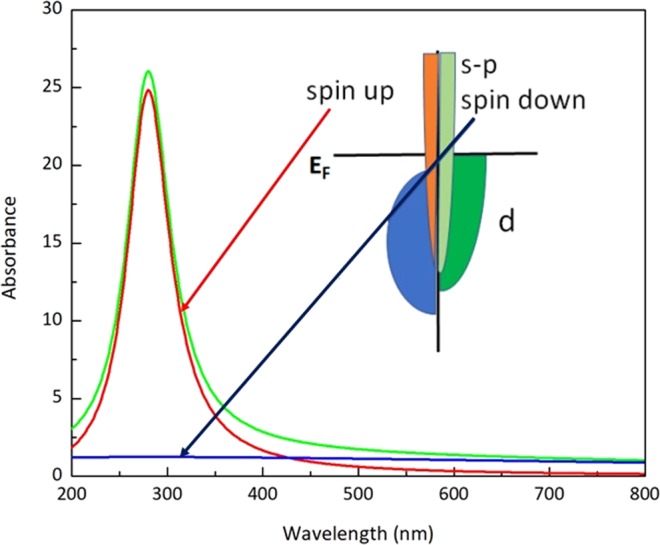
Two plasmon model for Co nanoparticles absorbance. ɷ_sp_ is taken to be 280 nm for wavelength, ${{\rm{\Gamma }}}_{\uparrow }\approx 6.55E+14\,rad/s$, ${{\rm{\Gamma }}}_{\downarrow }=20{{\rm{\Gamma }}}_{\uparrow }$. Green is the sum of red (spin up) and blue (spin down). Insert: a cartoon of the projected density of states typical for Co.

Thus, the absorption spectra should appear as a sharp resonance, due to spin-up electrons, plus a broad background coming from spin-down electrons (Fig. 2). Therefore, as soon as all possible electron scattering processes occur without spin-flip, meaning that two group of electrons are independent, one should expect sharp plasmon resonance. In particular, it requires single domain nanoparticles, since inter-domain walls increase probability of spin flip electron scattering and thus two group of electrons are no longer independent.

**Figure 3:**
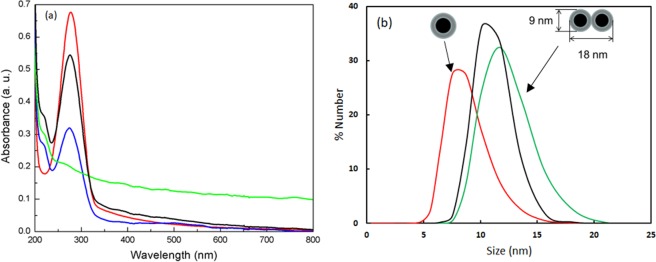
(**a**) Experimental absorbance of Co-NPs in hexane. As grown Co-NPs (red), after 1 hour sonication with external 130 mT DC magnetic field (blue), after 2.5 hours sonication with external 130 mT DC magnetic field (green, plasmon peak is demolished), no magnetic field and 1 hour sonication (black). (**b**) Co-NPs size distribution measured with dynamic light scattering (red)- as grown; (green) −2.5 hours sonication with external 130 mT DC magnetic field; (black) after 1 hour sonication without magnetic field.

To prove this concept, we perform experiments showing sharp plasmon resonance for isolated, single-domain Co nanoparticles (Co NPs), but plasmon resonance disappears if small, two-three particles aggregates are formed. The magnetization measurements by SQUID system (see SI.1) show superparamagnetic properties of the Co NPs at room temperature, which indicates the single-domain structure. The temperature dependence of the magnetization gives blocking temperature, which corresponds to the particle volume of this size. Below the blocking temperature field dependence of the magnetization has hysteresis behavior. All the results below correspond to the particles without an oxide shell (See magnetization loops and absorption spectrum in SI Fig. S1.8a,b). The SI Fig. S1.9 shows effect of oxidation on the magnetization loop for comparison. The shift of the hysteresis loop cooled to 10 K at field +1 T and opposite shift for the sample cooled at −1 T as in Fig. S1.9 allows to monitor the oxidation level of nanoparticles. Figure 3 demonstrates remarkable resonance quality of the representative spectrum for Co NPs in hexane solution. The plasmon resonance quality is about the same as for gold nanoparticles, which have resonance in the green spectral range (see comparison in SI Fig. S4.1). Co-NPs are isolated due to surfactants, trioctylphosphine and oleic acid. Dynamic light scattering data show an average size close to the mean size from TEM images.

The following experiment illustrates interaction of Co nanoparticles separated by thin surfactant shell. To initiate aggregation, the 130 mT DC magnetic field together with sonication were applied to the Co NP hexane suspension in a quartz cuvette. After 1 hour of “aggregation” the dynamic light scattering and absorption spectra were collected. Figure 3 shows reduced plasmon peak (shown in blue). After 2.5 hours of sonication in magnetic field, the plasmon peak disappeared (shown in green). The dynamic light scattering shown in the Fig. 3b gives increase in the hydrodynamic particles size from 8.7 to 12–13 nm corresponding to small, two-three particles aggregates. It is remarkable that the following sonication, without external magnetic field, separates aggregated particles and the plasmon resonance is restored. Thus, this magnetic/sonication induced aggregation is a reversible process.

**Figure 4:**
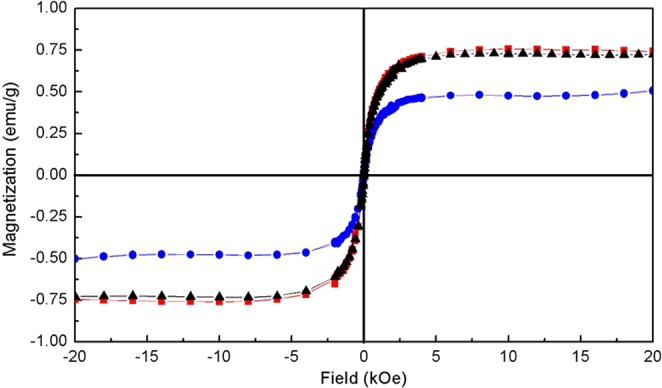
Magnetization of Co-NPs embedded in PMMA. Unaltered Co-NPs (red), after 2.5 hours sonication with external 130 mT DC magnetic field (blue), and 1 hour sonication with magnetic field off (black).

The magnetization of as-grown, post-aggregation, and post-sonication without magnetic field samples, shown in Fig. 4, also demonstrates a reversible behavior. It first decreases after 2.5 hours of sonication in magnetic field (Fig. 4 blue line), then returns to the initial value after sonication without magnetic field (Fig. 4 black line).

## Discussion

**Figure 5:**
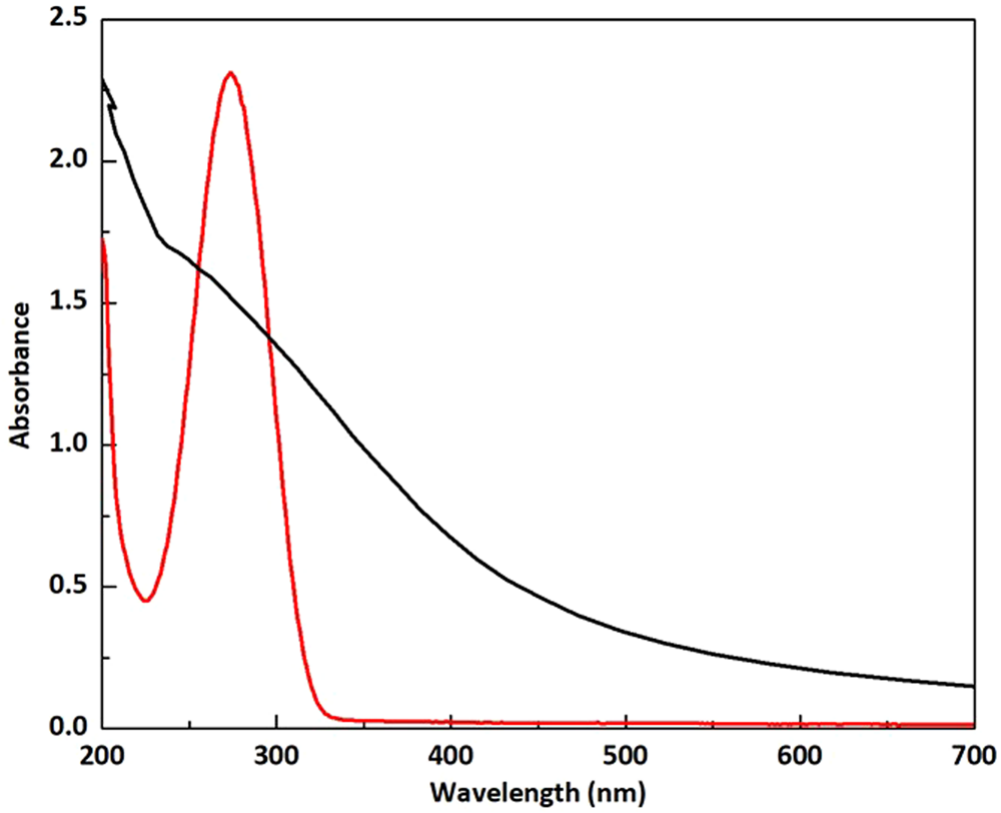
Absorbance of the Co NPs in hexane solution: experiment (red) and calculated (black) using J&C data^4^.

Note that calculated absorption spectrum for Co nanoparticles using J&C permittivity for Co films does not show a pronounced resonance (Fig. 5 black line) in contrast to the experimental spectrum for single-domain Co nanoparticles (Fig. 5 red line). For the nanoparticles with substantially sub-wavelength size, the dipole approximation reduces Mie’s theory to the following expression for the extinction cross-section^10^:

3 $${\sigma }_{ext}=9\frac{\omega {\varepsilon }_{h}^{1/2}}{c}V\frac{{\varepsilon }_{h}{\varepsilon }_{2}(\omega )}{{[{\varepsilon }_{1}(\omega )+2{\varepsilon }_{h}]}^{2}+{\varepsilon }_{2}^{2}(\omega )}$$

where $\omega$ is the light frequency, $V$ is the volume of the spherical particle, ${\varepsilon }_{h}$ is the dielectric permittivity of the surrounding (host) medium, and $c$ is the speed of light in vacuum. The spectrum of nanoparticles was calculated using bulk material complex permittivity $\varepsilon (\omega )={\varepsilon }_{1}+i{\varepsilon }_{2}$ from J&C^4^. Note that this approach for modeling nanoparticles’ spectra works for nonmagnetic metals like Au, Ag, but cannot be used for Co. One can see that the calculated spectrum using permittivity measured for Co films has no quality resonance. That is the reason why Co was not considered as a promising candidate so far. Indeed, if the film has multi-domain structure, where neighbour domains are typically disoriented, the electron scattering easily changes the spin polarization. Thus, electrons with spin-up become spin-down and immediately get largely increased relaxation rate due to available empty states in the d-band.

Note, there are sufficient number of publications showing the independence of two channel of conductivity. The first-principle simulations give the mean free path of electrons for spin up (12 nm) and spin down (0.6 nm) calculated for Co (please see refs.^6,7^ and references there). According to the literature on giant magnetoresistance the strongly different mean free path for spin-up and spin down electrons is well established fact. Indeed, the “two currents” conduction concept proposed by N. Mott^2,3^ and used by Fert and Campbell^11^ to explain specific behaviors in the conductivity of usual ferromagnetic metals Fe, Ni, Co, and their alloys. Because of the strong exchange interaction favouring parallel orientation of electron spins, the “spin up” and “spin down” 3d bands are shifted in energy. The spin conserving s-to-d transitions are the main source of s electron scattering. This has two major consequences on transport: firstly, the spin imbalanced density of states for 3d electrons at Fermi energy results in strongly spin dependent scattering probabilities, and, secondly, between two spin flip scattering events an electron can undergo many scattering events while keeping the same spin direction. Thus at the limit where spin-flip scattering events are negligible, conduction happens in parallel through two spin channels that have very different conductivities. The spin dependent scattering probability results in very different mean free paths ${\lambda }_{\uparrow }$ and ${\lambda }_{\downarrow }$, or equivalently relaxation times ${\tau }_{\uparrow }$ and ${\tau }_{\downarrow }$. In usual thin metallic layers the ${\lambda }_{\uparrow }$/${\lambda }_{\downarrow }$ ratios can be highly variable^12–14^. The discovery of the giant magnetoresistance^3,13^ was actually able to show evidences of the spin dependent electron transport.

Here a concept of the possibility to have two independent plasmons explains the observed behavior, namely sharp plasmon in contrast to J&C based predictions and the vanishing due to mixing of these two channels. Indeed, according to simulations performed for GMR a number of s-p electrons in Co is approximately equal for spin-up and spin-down electrons^6^. Terms majority and minority electrons and the bands splitting due to exchange interaction is relevant to d-electrons. This splitting makes difference in the density of empty states in the d-band and thus providing conditions for strongly different mean free path of the s-p electrons. The plasma frequency is related to the conduction electrons. Taking into account that the ratio between spectral width of two plasmons can be up to 20, certainly there is an overlap and the sharp plasmon has the same frequency as a spectral portion of the broad plasmon. Note, that since the plasmon is a collective excitation of the conduction electrons in s-p band their resonance quality factor is governed by the electron relaxation processes, thus related to the conductivity. Regarding the reason for the vanishing of the plasmon mode resonance under aggregation, induced by magnetic field with sonication and then reversed by sonication, this can be explained only by the electron scattering with spin flip in the presence of second magnetic particles. Indeed, the spin-flip scattering is the only difference relative to the Ag, Au or other nonmagnetic nanoparticles. Note that calculation of the probability of scattering with spin-flip is a difficult theoretical task. The exsisting literature contains only a qualitative result that the spin flip becomes significant in the presence of another domain and depends on several parameters, which are hardly detectable. Our data provides an opportunity to study these processes. Indeed, usual method to characterize the relaxation constants is to measure a spectral width of the plasmonic resonance. Another method is based on the time resolved response dynamics of plasmon excitation^15^, where ultrafast studies of plasmons in Co nanoparticles, including relaxation times, have been reported.

**Figure 6:**
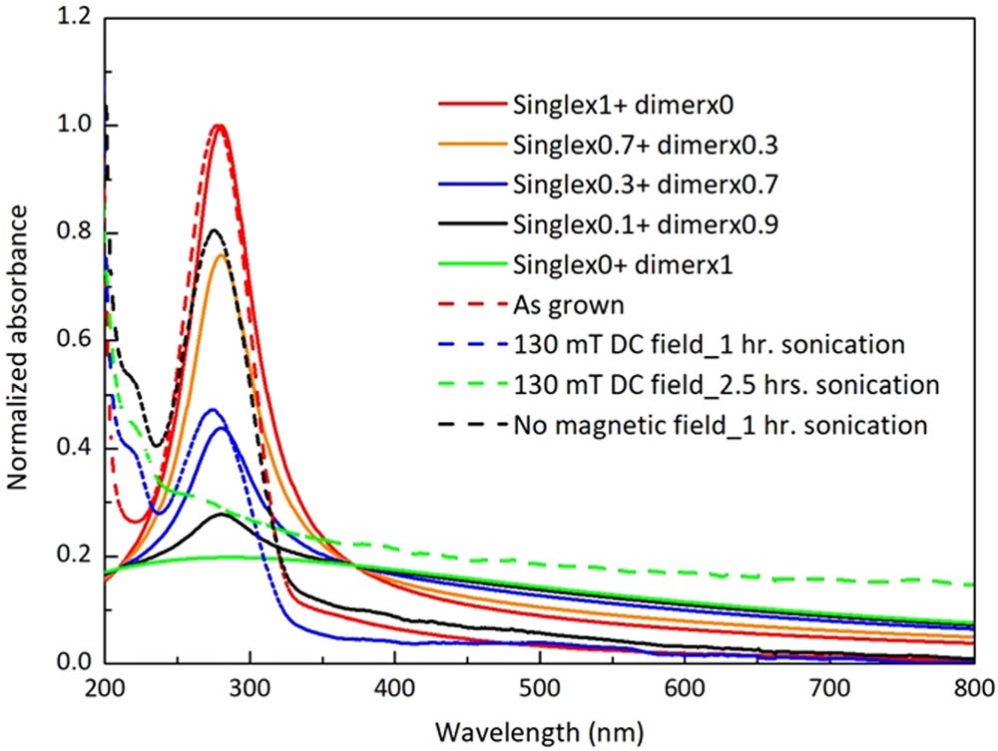
Calculated absorption spectra, combined as a sum of monomers and dimers of cobalt nanoparticles with different portion of nanoparticles involved in dimers: red- 0, yellow - 0.3, black - 0.7, green - 0.9, blue - 1. Relaxation constants for single nanoparticles ${{\Gamma }}_{\uparrow }=6.55E+14\,rad/s,$ ${{\rm{\Gamma }}}_{\downarrow }=20{{\rm{\Gamma }}}_{\uparrow }$, for dimer the relaxation constant in both particles is ${{\Gamma }}_{eff}=9{{\Gamma }}_{\uparrow }$. Dash lines spectra correspond to experimental ones from Fig. 3.

The inter-particle dipole-dipole interaction makes an important difference between plasmons in Au (or other nonmagnetic materials) and in Co. The dipolar coupling itself between nanoparticles can keep the resonance strong. What is specific for Co is the probability of electron scattering with spin-flip, which can mix the two independent groups and obviously always reducing the relaxation time of the electrons involved in the sharp resonance plasmon oscillations. The experimental results in Fig. 3a can be modeled in the following way. Imagine, that during aggregation you have gradually decreasing number of isolated particle, and increasing the number of aggregates. Along with this process, any aggregate just gets strongly increased relaxation constant due to spin-flip, so that it manifests in a broad and week absorption. Thus the frequency shift is hardly visible. The remaining portion of isolated particles still show resonance at the same frequency. Here we illustrate this point with the calculations for a dimer of particle placed close (called dimer) and far (called single on the Fig. 6 below) to each other. The model implies that the aggregation results not only in dipole-dipole interaction but in strong change of the relaxation constant due to electron scattering with spin-flip. Thus the effective relaxation constant with spin-flip can be in between the initial relaxation constants ${{\Gamma }}_{\uparrow } < {{\Gamma }}_{eff} < 20{{\Gamma }}_{\uparrow }$. The different spectra show different proportion between single nanoparticles and dimers. Thus the calculated data based on the model of spin-polarized NPs show similar spectra with experimental ones in Fig. 3 and reproduced in Fig. 6 for comparison.

The spectra in Fig. 6 were calculated following ref.^16^. *For a single particle (monomer)*,

4 $$Im(\frac{d}{E})=\frac{{a}^{3}{\delta }_{1}}{{X}^{2}+{\delta }_{1}^{2}}$$

Here, the radius of a particle $a=4.35\,{\rm{nm}}$. The half-width of the resonance at half height ${\delta }_{1}=\frac{2\omega {{\rm{\Gamma }}}_{\uparrow }}{{\omega }_{sp}^{2}}$. The relaxation constant for spin-up ${{\Gamma }}_{\uparrow }=6.55E+14\,{\rm{rad}}$ s^−1^ = 0.431 eV, ${\omega }_{sp}=2\pi {f}_{sp}=\frac{2\pi c}{{\lambda }_{sp}}$=6.728E + 15 rad s^−1^; ${\lambda }_{sp}=280\,{\rm{nm}},$ $\frac{{{\rm{\Gamma }}}_{\uparrow }}{{\omega }_{sp}}=0.097$. The absorbance

5 $$Ab=\frac{N(2\pi /\lambda )Im(\frac{d}{E})L}{\mathrm{ln}\,10},$$

where the density of particles (N) = 6.03E + 20 m^−3^, path length (L) = 0.01 m, ln 10 = 2.302.

For a dimer with electric field parallel to the dimer axis,

6 $$Im(\frac{{d}_{n}}{{E}_{n}})=\frac{({a}_{1}^{3}+{a}_{2}^{3})\delta }{1+A}\frac{{(X-2\xi )}^{2}+{\delta }^{2}+A[{(X+2\xi )}^{2}+{\delta }^{2}]}{[{(X+2\xi )}^{2}+{\delta }^{2}][{(X-2\xi )}^{2}+{\delta }^{2}]}.$$

Here, $\delta =\frac{2\omega {{\Gamma }}_{eff}}{{\omega }_{sp}^{2}}$, for dimers ${{\Gamma }}_{eff}=9{{\Gamma }}_{\uparrow }=5.9E+15\,{\rm{rad}}$ s^−1^ = 3.9 eV, $\frac{{{\rm{\Gamma }}}_{eff}}{{\omega }_{sp}}=0.88,$ radii of the particles, ${a}_{1}={a}_{2}=4.35\,{\rm{nm}}$, $a={({a}_{1}{a}_{2})}^{1/2}$ = 4.35 nm, $\xi ={(a/\rho )}^{3}$ = 0.125, where, ρ is the distance between the centers of the particles. Finally, the amplitude $A=\frac{{({a}_{1}^{3/2}-{a}_{2}^{3/2})}^{2}}{{({a}_{1}^{3/2}+{a}_{2}^{3/2})}^{2}}=0$, and $X=\frac{{\omega }^{2}}{{\omega }_{sp}^{2}}-1$. The absorbance is calculated also for a dimer with electric field perpendicular to the dimer axis following Eq. (7) and same notations as near Eq. (6), then an average is taken.

7 $$Im(\frac{{d}_{\perp }}{{E}_{\perp }})=\frac{({a}_{1}^{3}+{a}_{2}^{3})\delta }{1+A}\frac{A[{(X-\xi )}^{2}+{\delta }^{2}]+{(X+\xi )}^{2}+{\delta }^{2}}{{[X+\xi )}^{2}+{\delta }^{2}][{(X-\xi )}^{2}+{\delta }^{2}]}.$$

**Figure 7:**
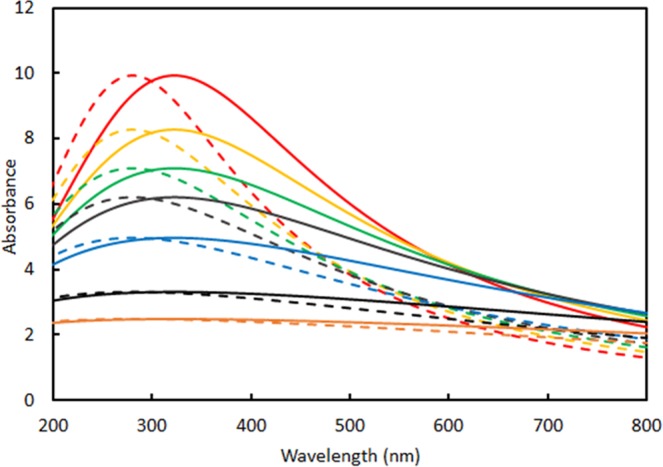
Calculated absorption spectra of the dimer with distance between centers 8.75 nm (solid lines) and 90 nm (dash lines) at different ${{\rm{\Gamma }}}_{eff}$. Polarization is parallel to the dimer axis. ${{\Gamma }}_{eff}\,=5{{\Gamma }}_{\uparrow }\,(red)$, ${{\Gamma }}_{eff}=6{{\Gamma }}_{\uparrow }(yellow)$, ${{\Gamma }}_{eff}=7{{\Gamma }}_{\uparrow }\,(green)$, ${{\Gamma }}_{eff}=8{{\Gamma }}_{\uparrow }(brown),\,{{\Gamma }}_{eff}=10{{\Gamma }}_{\uparrow }(blue),\,{{\Gamma }}_{eff}=15{{\Gamma }}_{\uparrow }(black),\,{{\Gamma }}_{eff}=20{{\Gamma }}_{\uparrow }(orange)$. ${\rm{Spectral}}\,{\rm{half}}\,{\rm{width}}\,{\rm{at}}\,{\rm{half}}\,{\rm{hight}}\,({{\Gamma }}_{\uparrow })=6.55E+14\,rad$s^−1^.

We model the case when the aggregation results not only in dipole-dipole interaction but in strong change of the relaxation constant due to electron scattering with spin-flip. The spin-flip scattering results in mixing of two channels resulting in some effective relaxation constant, which can be in the range between spin up and spin down relaxation constants for isolated nanoparticles. Figure 7 shows comparison of monomers (dash lines) and dimers (solid lines) for different effective relaxation constants.

In summary, cobalt nanoparticles synthesized by high temperature reduction of cobalt salt show strong plasmon resonance at 280 nm with better quality than that of gold nanoparticles in the visible spectrum. Our experiments with Co nanoparticles clearly show a new type of plasmon excitation, which is specific for spin polarized single domain nanoparticles. This type of plasmon has unusual properties due to existence of two independent groups of electrons with opposite spins providing weak interaction so that all electron scattering processes occur without spin flip. Magnetic response of the nanoparticles enables controlled and reversible aggregation accompanied by the tailoring of optical absorption.

These conclusions are supported by the following arguments. An extensive collection of literature^6,7^ on GMR including first principle simulations proof that the materials with spin polarization of d-electrons have two independent channels of the conductivity (note, that the conductivity is mainly caused by the s-p electrons). The values of conductivity are very different for two channels. The two channels are independent only if the electron scattering goes without spin-flip. The scattering with spin flip can be initiated by the domain structure.

These three points, known from the Mott’s seminal papers, allow explaining our experimental observations, which were possible only by following the described synthesis recipe.

Our observations include correlation of sharp plasmon resonance with magnetic properties, specifically only singe-domain superparamagnetic nanoparticles show sharp resonance. Larger nanoparticles with domain structure does not show that. The optical absorption measurements are accompanied by the transmission electron microscopy, dynamic light scattering, and magnetometry to characterize the domain structure in a single nanoparticle, aggregation state, and magnetization. The vanishing of the resonance under aggregation (induced by magnetic field with sonication and reversible) can be explained only by the electron scattering with spin flip in the presence of second magnetic particles, since the spin-flip scattering is the only difference relative to the Ag or Au nanoparticles.

## Methods

### The SEM EDX image of the cobalt nanoparticles synthesized by the high temperature decomposition of cobalt salt

We observed the large aggregates of cobalt nanoparticles instead of isolated particles because the circular magnet placed underneath the silicon substrate attracted the magnetic nanoparticles from the solution. An energy dispersive X-ray (EDX) spectrum analysis as shown in section S3 of the SI has been obtained from the cobalt nanoparticle sample. This spectrum clearly shows the presence of cobalt peaks. In addition, EDX spectrum also shows the presence of nickel peaks because the sample was made conductive by coating it with nickel.

### SQUID magnetic measurements

Magnetic properties of cobalt nanoparticles embedded into PMMA host matrix were measured using 7 Tesla SQUID magnetometer (Magnetic Property Measurement Device, Quantum Design Inc.). The detailed analysis is presented in the S1 of SI. Co NPs were dispersed in PMMA, deposited on a substrate, dried, then the PMMA film with embedded Co NPs were packed in capsule as shown in Fig. S1.1 of the SI.

## Supplementary information

New mechanism of plasmons specific for spin-polarized nanoparticles
