# Supplementary material for: New mechanism of plasmons specific for spin-polarized nanoparticles
Source: Sci Rep. 2019 Feb 14;9:2019. doi: 10.1038/s41598-019-38657-w (PMC6375939; doi:10.1038/s41598-019-38657-w)
Supplement: Supplementary file 1 — New mechanism of plasmons specific for spin-polarized nanoparticles [file 41598_2019_38657_MOESM1_ESM.pdf]

Supporting Information

New mechanism of plasmons specific for spin-polarized nanoparticles

Hari L. Bhatta[1], Ali E. Aliev[2], and Vladimir P. Drachev*[1,3]

[1] Department of Physics and Advanced Materials and Mechanical Processing Institute,

University of North Texas, Denton, TX 76203, USA

[2] A. G. MacDiarmid NanoTech Institute,

University of Texas at Dallas, Richardson, TX 75083, USA

[3]Skolkovo Institute of Science and Technology, Moscow 121205, Russia

*vladimir.drachev@unt.edu

The Supporting Information is organized in the following sections:

S1. Magnetic properties of Co nanoparticles.
S2. Dynamic light scattering.
S3. Structural characterization: SEM Image and EDX spectrum; TEM Image and size distribution graph; High angle annular dark field (HAADF) micrograph.
S4. Long term variation in absorption spectra of Co nanoparticles suspended in hexane.
S5. Absorption cross-section spectra of Co nanoparticles in comparison with Au and Ag nanoparticles.

S1: Magnetic properties of Co nanoparticles.

Magnetic properties of cobalt nanoparticles embedded into a PMMA host matrix were measured using the DC scan technique of 7 Tesla supermagnet SQUID magnetometer (Magnetic Property Measurement System, Quantum Design Inc.). Co NPs were dispersed in PMMA, deposited onto a substrate, dried, and then the PMMA film with embedded Co NPs were packed into a gelatin capsule. In order to reduce the influence of the shape (demagnetization effect), all Co NPs-embedded sheets were placed into elongated gelatine capsules aligned parallel to the applied magnetic field.

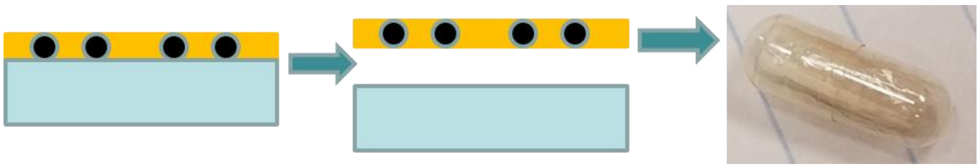

Figure S1.1. Fabrication steps for magnetic measurements.

The temperature dependence of magnetization in field cooling (FC) and zero field cooling (ZFC) regimes were exploited to determine the blocking temperature (T_b) at which the ZFC magnetization shows a pronounced peak. T_b is the temperature below which the single domain nanoparticles shows hysteresis loop in the magnetization and above T_b shows superparamagnetic properties[1]. The observed curves for FC and ZFC regimes shown in Fig. S1.2 (a) are typical for ferromagnetic nanoparticles.

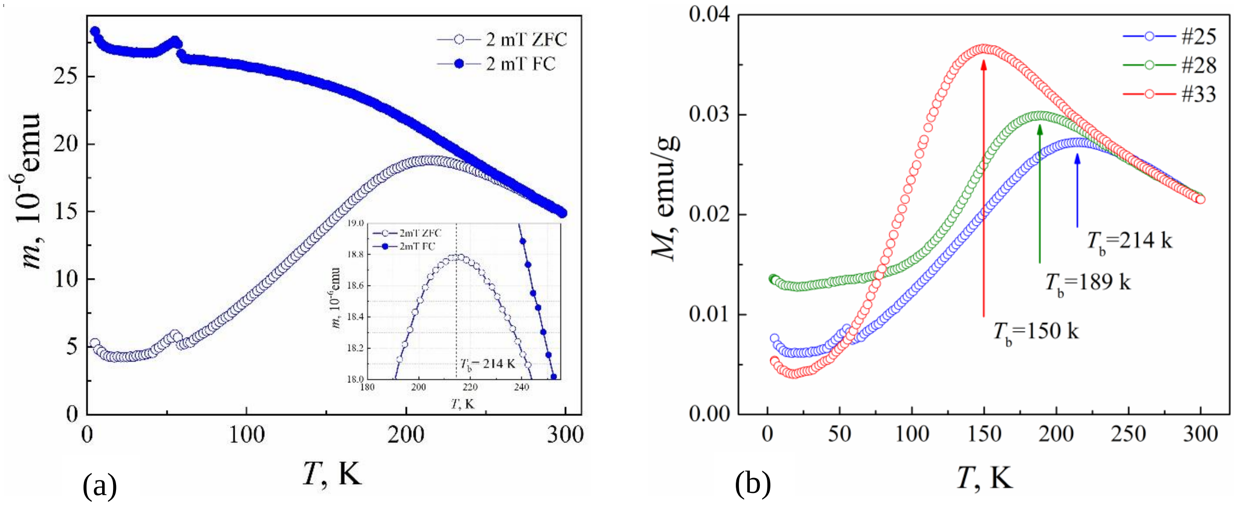

Figure S1.2. (a) Temperature dependence of magnetic moment measured in FC (solid blue circles) and ZFC (open blue circles) regimes at applied magnetic field of 2 mT (sample #25). The inset shows an expanded view of the peak for ZFC magnetization. The small bump at 52 K indicates an oxygen contents in the sealed capsule. (b) Shift of ZFC magnetization peaks towards lower temperatures for decreasing particle sizes: 9.5 nm for sample #25; 7.6 nm for sample #28; and 6.6 nm for sample #33, respectively.

For very small particles at finite temperatures (T_b) the anisotropy energy becomes comparable to the thermal energy resulting in superparamagnetic (SP) relaxation. The SP blocking temperature (T_b) is defined as the temperature at which the SP relaxation time (response of magnetic dipole) equals the timescale of the experimental technique used to study the magnetic properties, $\omega\tau = 1$. The SP relaxation time τ given by following equation,[2]

$$\tau = \tau_0 \exp\left(\frac{K_u V}{k_b T}\right), \qquad \text{(S1.1)}$$

was measured using AC Measurement System (ACMS) option of Physical Property Measurement Device (Quantum Design Inc.) at frequencies in the range of $10 \le f \le 10^4$ Hz with alternative field amplitude $H_{AC} = \pm$ 1mT. Here K_u is the magnetic anisotropy constant, V is the particle volume, k_B is Boltzmann's constant and T is the temperature. The value of τ_0 extracted from the linear extrapolation of τ from zero to 1000/T is $3.8\text{x}10^{-14}$ s. Hence, for DC measurements, where $\ln(\tau/\tau_0) = 29$, the blocking temperature, above which the particle become a single domain magnet and is small enough to display superparamagnetism, should roughly satisfy the relationship

$$T_b \approx \frac{K_u V}{29 k_B}. \qquad \text{(S1.2)}$$

With a knowledge of average particle diameter from the precise analysis of TEM images ($d_{\#25}$ = 9.5 nm, $d_{\#28}$ = 7.6 nm, $d_{\#33}$ = 6.6 nm,) and AC and DC magnetic measurements the extracted magnetic anisotropy constants ($K_u^{\#25} = 2.74\text{x}10^6$ erg/cm^3, $K_u^{\#28} = 3.73\text{x}10^6$ erg/cm^3, $K_u^{\#33} = 3.9\text{x}10^6$ erg/cm^3) fall between bulk fcc and hcp structures ($2.7\text{x}10^6$ erg/cm^3 for fcc and $4.7\text{x}10^6$ erg/cm^3 for hcp, respectively). The increase in anisotropy constant with decrease in particle size is due to surface anisotropy. The fraction of Co atoms on the surface of nanoparticles increases with decrease in particle size, which results in a large anisotropy constant, K_u.[3]

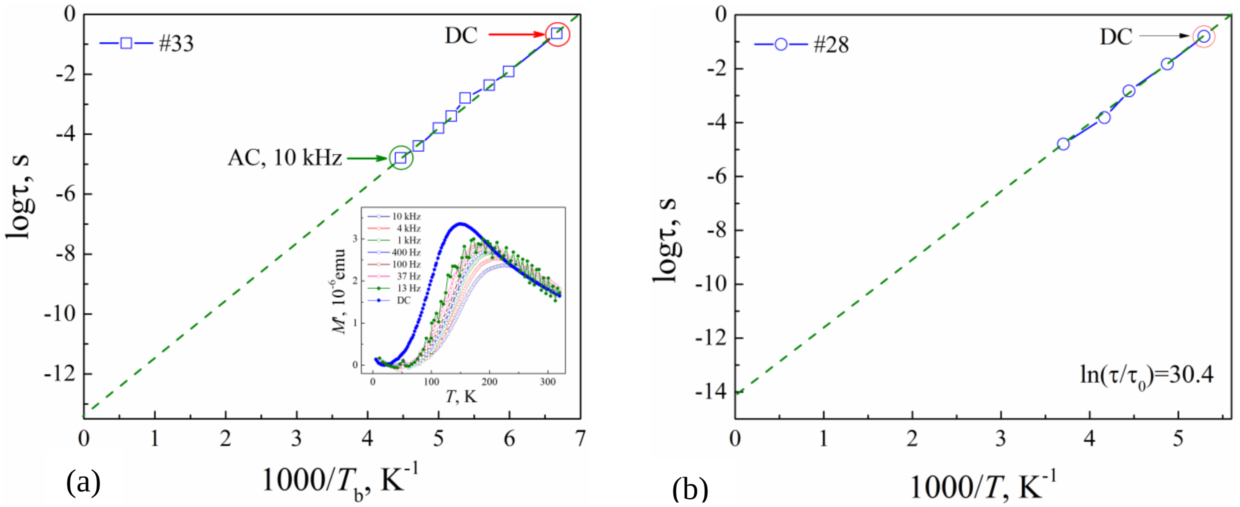

Figure S1.3. Thermal variation of the relaxation time for sample #33 (a) and sample #28 (b). The green dashed line is an extrapolation to 1000/T_b = 0. The inset to (a) shows the ZFC magnetization measured at different frequencies.

Above the blocking temperature, the susceptibility is roughly linear with temperature (see Figure S1.4) in accord with the Curie law, $\chi \sim C/T$, where $C = N\mu_0\mu^2/k_B$ is the Curie constant. This linearity also implies a low interaction amongst the nanoparticles.

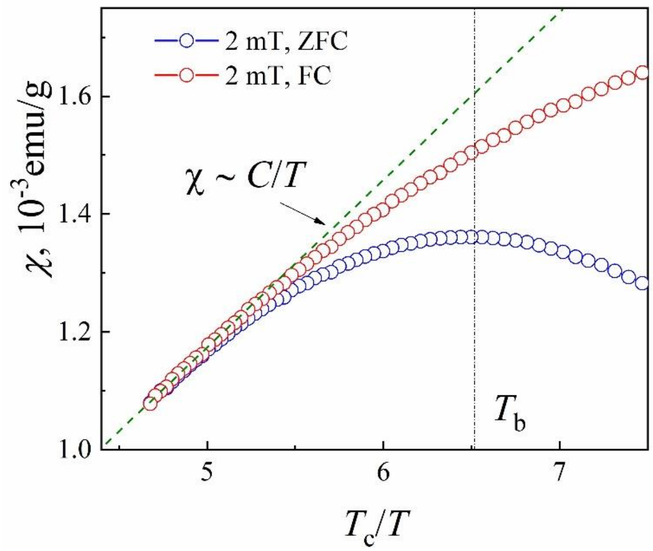

Figure S1.4. The FC and ZFC magnetization plotted versus reciprocal temperature, T_c = 1394 K is the Curie temperature for bulk Co.

On the other hand, the increase of particle size should shift the anisotropy constant towards the balk value (2.7×10^6 erg/cm^3).[3] Figure S1.5 (b) shows that for particles larger than 8 nm the anisotropy energy is close to the bulk fcc value, 2.7×10^6 erg/cm^3. This reduces the estimated particle size to ~ 8.1 nm.

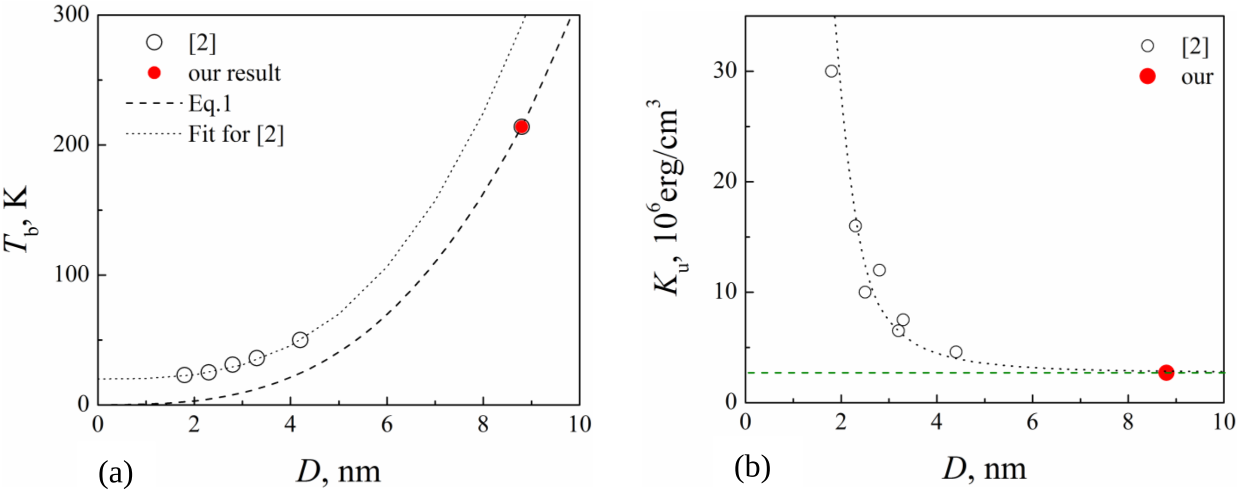

Figure S1.5. (a) The particle diameter dependence of blocking temperature. Dotted and dashed curves show a fitting to the cub (volume) dependence. (b) The particle diameter dependence of anisotropy energy. Dot reciprocal-cub line is a fitting of Ref.[2] data. The bulk value for fcc cobalt shown by dashed green line.

The high-field magnetization behaviour of the Co-nanoparticles for 5, 100 and 298 K are shown in Figure S1.6. At 5 K the particles exhibit no-saturation behaviour up to highest available field of 7 T. The saturation magnetization obtained from the extrapolation of $1/H \rightarrow 0$ shows value (~ 10 emu/g) close to that obtained in Ref. [4] for 7 nm cobalt nanoparticles having fcc structure. At the same time, this value is one order lower than bulk fcc Co (166 emu/g or 175 emu/g, see in Ref. [5]). The reduced remanence, M_r/M_s = 0.02, is far below of theoretical values for nanocrystals having uniaxial anisotropy, such as cobalt, in the hcp form (0.5) or in the fcc form (0.8).

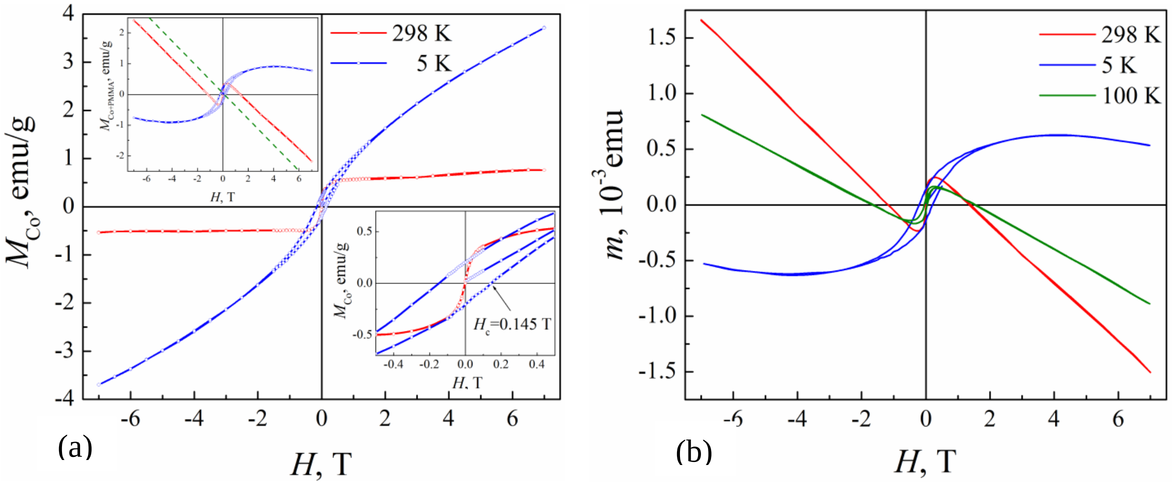

Figure S1.6. (#25). (a) Field dependence of magnetization of Co nanoparticles (0.69 mg) dispersed in 10.31 g PMMA taken at 5 K (open blue circles) and 298 K (open red circles). Main panel shows magnetization of Co-nanoparticles with subtracted diamagnetic contribution of PMMA host material. Top-left inset shows as-measured magnetization at 5 and 298 K. Green dashed line shows the reference line for diamagnetic contribution of host material. The bottom-right inset shows an expanded view of magnetization at low fields. (b) Field dependence of magnetic moment for three temperatures: 5, 100, 298 K.

Among the possible explanations for the reduced magnetization at saturation is the exchange coupling between adjacent particles. The dipole coupling enhancements are attributed to the long-range order of

the 2D lattice (particles are embedded into thin PMMA film) and collective “flips” of the magnetic dipoles. On the other hand, the coercive field at T = 5 K, is relatively high, H_c = 0.145 T.

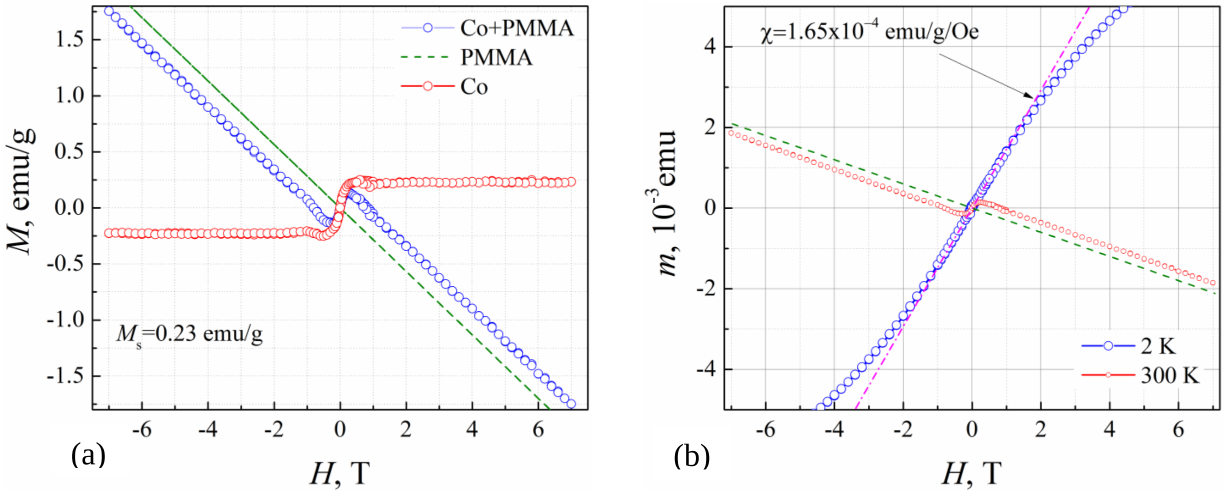

Figure S1.7. (#33). (a) Field dependence of magnetization of Co nanoparticles (1.06 mg) dispersed in PMMA taken at 300 K (open blue circles) and magnetization of Co-nanoparticles with subtracted diamagnetic contribution of PMMA host material (open red circles). Green dashed line shows the reference line for diamagnetic contribution of host material. (b) Field dependence of magnetic moment for two temperatures: 2 K and 300 K.

The magnetic moment per particle (#25_1L) was calculated from the susceptibility χ above the blocking temperature at low field using Eq. (S1.3)[5].

$$\chi = \frac{M_s \mu}{3k_b T} \qquad \text{S1.3}$$

where μ is the magnetic moment per particle, M_s is the saturation magnetization, and k_b is Boltzmann's constant. The diamagnetic susceptibility of the PMMA matrix was measured and subtracted to obtain these results (see Fig. S1.7): At 214 K the contribution of the diamagnetic part (PMMA) for sample #25 is $0.45\text{x}10^{-6}$ emu; Magnetic moment, $m = (18.8 - 0.45)\text{x}10^{-6}$ emu = 18.35 $\text{x}10^{-6}$ emu; Saturation magnetization M at T = 298 K (the same at 200 K): M_s = 0.57 emu/g. Susceptibility χ measured for applied field H = 2 mT at 214 K: $\chi = m/(m_\rho \cdot H) = 18.35\text{x}10^{-6}/(0.69\text{x}10^{-3}$ x 2 mT) = $1.33\text{x}10^{-7}$ emu/g·T. Here m_ρ = 0.69 mg is the mass of Co nanoparticles in PMMA host material (10.31 mg). Then, the magnetic moment per particle above T = 214 K:

$$\mu = \frac{3k_B T\chi}{M_s} = \frac{3\cdot 1.38\cdot 10^{-16}\cdot 214\cdot 1.33\cdot 10^{-3}}{0.57} \cong 2.07\cdot 10^{-16} \text{ Erg/G}$$

Number of Bohr magnetons: $n = \mu/\mu_b = 2.07\text{x}10^{-16}/(9.27\text{x}10^{-21})$ = 22300, which is ~ 0.687μ_b per atom. Number of Co atoms in single particle n_a: $m_{(\text{Co atom})}$ = mol. weight/N_A = 58.933 g/mol/$6.022\text{x}10^{-23}$ atom/mol = $9.786\text{x}10^{-23}$ g/atom. Thus, $n_a = m_{\text{particle}}/m_{(\text{Co atom})} = 3.175\text{x}10^{-18}/9.786\text{x}10^{-23}$ = 32444. The above number of Bohr magnetons per atom in a single 8.8 nm nanoparticle is much lower than the number of Bohr magnetons per single atom in bulk cobalt, 1.73 (hcp).

Oxidation shell monitoring

Co nanoparticles coated with CoO should exhibit exchange anisotropy due to interfacial interaction between ferromagnetic Co metal and antiferromagnetic CoO. The presence or absence of the oxidation shell can be evident from magnetization loops taken at low temperature and cooled at nonzero field. Relatively fresh samples measured within 1 - 3 weeks do not show any shift of hysteresis loop (Fig.S1.8a) and good quality of the absorbance resonance (Fig. S1.8b). For comparison Figure S1.9 shows oxidation case. The shift of hysteresis loop, taken at low temperatures (10 K) after the sample was cooled in a field of 1 T or -1T, implies that CoO was formed on the particle surfaces (Figure S1.9). These measurements were taken for a sample after 4 months since the preparation date. The samples were embedded into PMMA matrix, the oxidation, perhaps, comes from a surfactant shell on the surface of Co-nanoparticles.

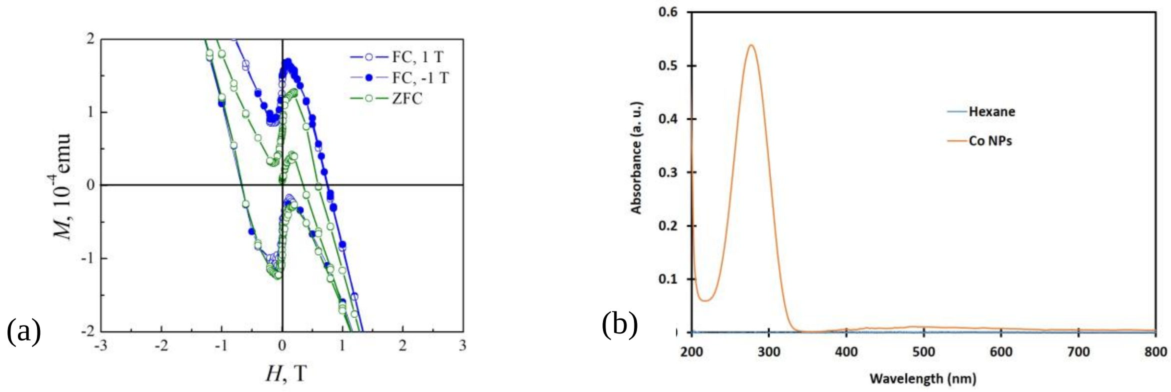

Figure S1.8. (a) No shift of magnetization loops for sample cooled in field + 1 T (open blue circles), -1 T (filled blue circles), and cooled at zero field (open green circles). (b) The absorbance spectrum for the same sample collected for CoNPs in hexane solution (brown) and hexane (blue).

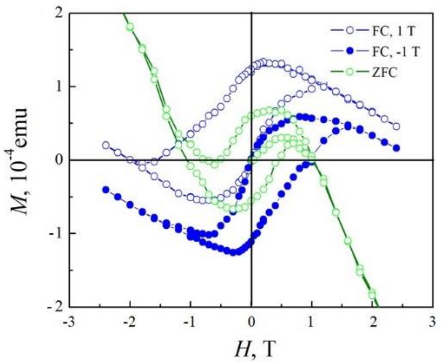

Figure S1.9. Shift of magnetization loops for sample cooled in field + 1 T (open blue circles) and -1 T (filled blue circles). The magnetization loop for the sample cooled in zero field is symmetrical (open green circles).

S2: Dynamic light scattering.

Shown in Figure S2.1 are dynamic light scattering (DLS) results on particle size distribution of undiluted Co nanoparticles samples. As-grown Co nanoparticles have DLS average particle size 8.7 nm. Then, the solution was sonicated in the presence of 130 mT external DC magnetic field. The effective particle size has been increased from 8.7 nm to 12 nm. This increase in particle size is due to agglomeration of Co nanoparticles in the presence of magnetic field. To reverse the agglomeration, the sample was sonicated for 1 hour in the absence of an external DC magnetic field. The particle size has been reduced to 10 nm. When an external field is removed, sonication isolates the nanoparticles and results in decreasing of the effective particle size. The DLS measurements were performed at the same time as the collection of the optical spectra and the sample was prepared for magnetometry like in Figure S1.1 (the results are shown in the main text).

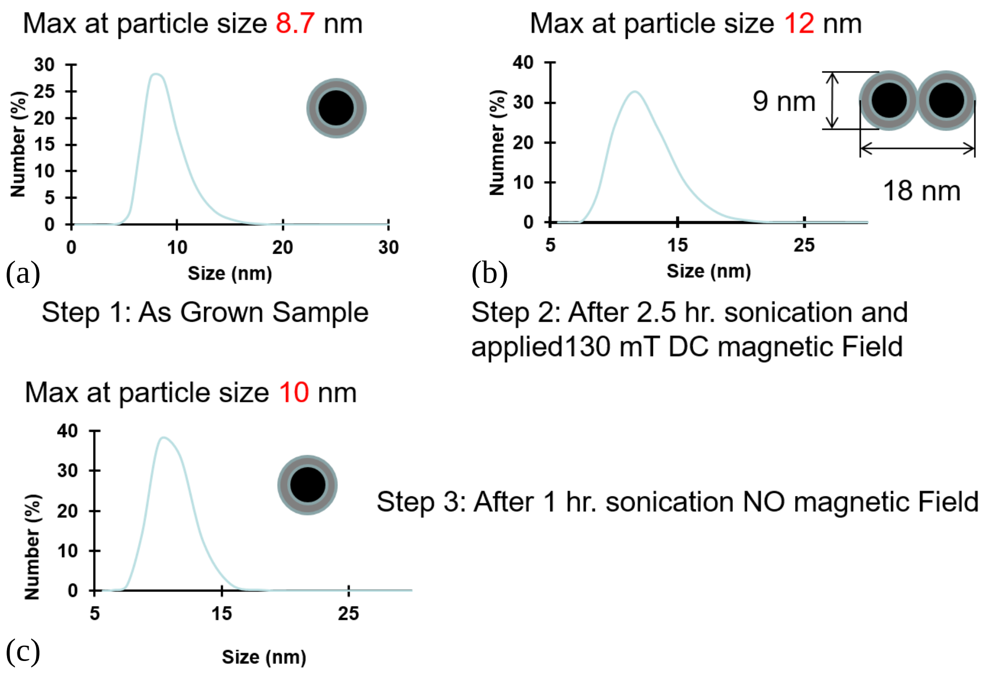

Figure S2.1. Dynamic light scattering (DLS) size distribution of Co nanoparticles (a), as grown sample; (b), after 2.5 hours sonication with external 130 mT DC magnetic field; and (c), after 1 hour sonication without external magnetic field.

S3: Structural characterization.

SEM image and EDX spectrum of Co nanoparticles.

Figure S3.1 shows SEM image of the cobalt nanoparticles prepared by the method of high temperature reduction of cobalt salt and corresponding EDX spectrum. SEM image shows the large aggregates of Co nanoparticles. EDX spectrum shows the presence of Co peaks. In addition, EDX spectrum also shows the presence of nickel peaks because the sample was made conductive by coating with nickel.

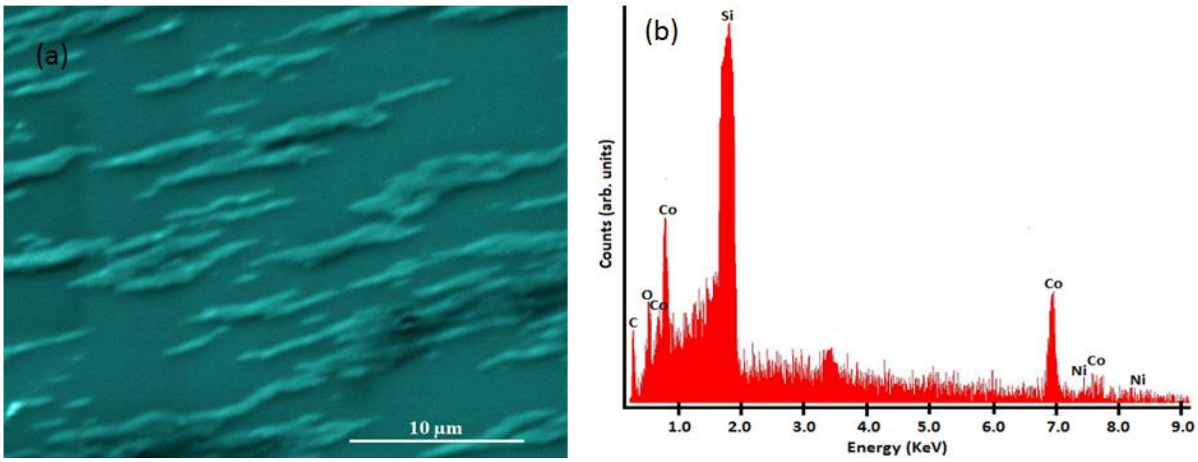

Figure S3.1. **(**a) SEM image of the Co nanoparticles. (b) Corresponding EDX spectrum.

TEM image and size distribution graph of Co nanoparticles.

Figure S3.2 shows TEM images and a size distribution graph of Co nanoparticles. Co nanoparticles are self-assembled and separated by an organic ligand shell. The inset in Figure S3.2(a) shows a high resolution TEM image of a spherical cobalt nanoparticle. The size distribution graph of Co nanoparticles shows the particle size ranges from 6 - 12 nm with average particle diameter of 8.7 nm.

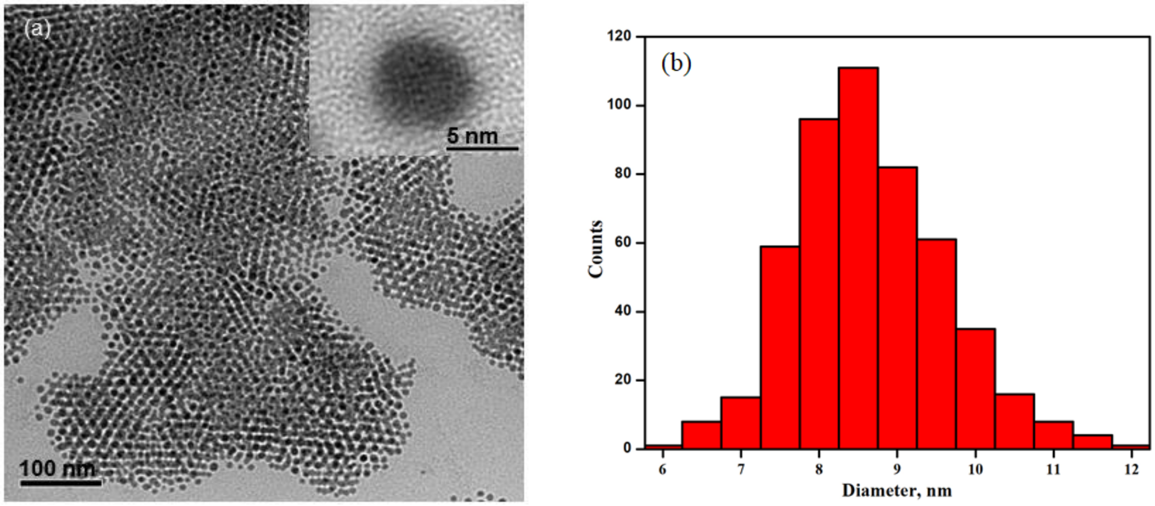

Figure S3.2. **(**a**)** TEM image of Co nanoparticles. Top-right inset shows a high magnification TEM image of a spherical single cobalt nanoparticle. (b**)** Size distribution graph of Co nanoparticles.

High angle annular dark field (HAADF) micrograph of Co nanoparticles.

Figure S3.3 shows the high angle annular dark field (HAADF) scanning transmission electron microscopy (STEM) image of cobalt nanoparticles. HAADF image proves that all the particles are made from the same material, cobalt.

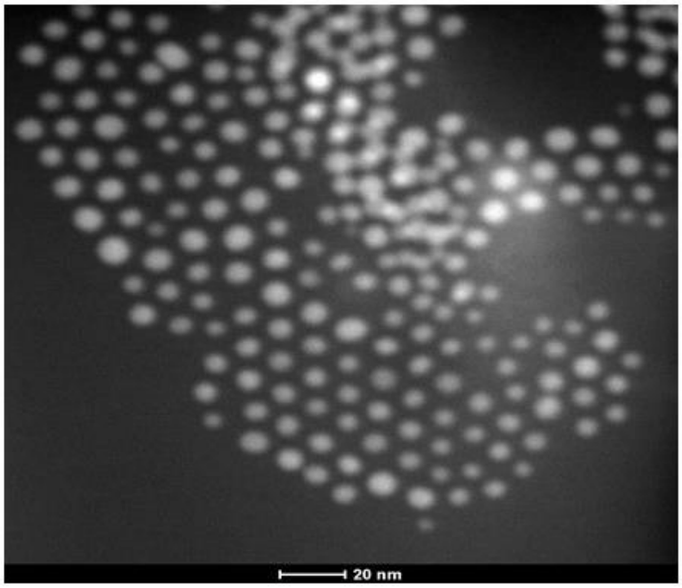

Figure S3.3. High angle annular dark field (HAADF) micrograph of Co nanoparticles.

S4: Long-term variations of absorption spectra for Co nanoparticles suspended in hexane.

Figure S4.1 shows the time-dependent UV-Visible absorption spectra of Co nanoparticles. No red shift in the peak position confirms the colloidal stability of Co nanoparticles.

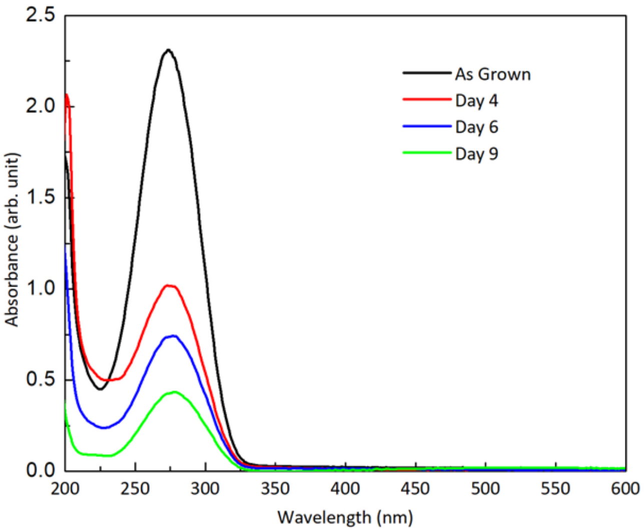

Figure S4.1. Time variation of absorption spectra for Co nanoparticles suspended in hexane.

S5: Absorption cross-section spectra of Co nanoparticles in comparison with Au and Ag nanoparticles.

Figure S5.1 shows the absorption cross-section for Co nanoparticles (red, experiment, this work) and Au (blue) and Ag (green) nanoparticles calculated using J&C data[1]. The calculation method is the same as in the main text.

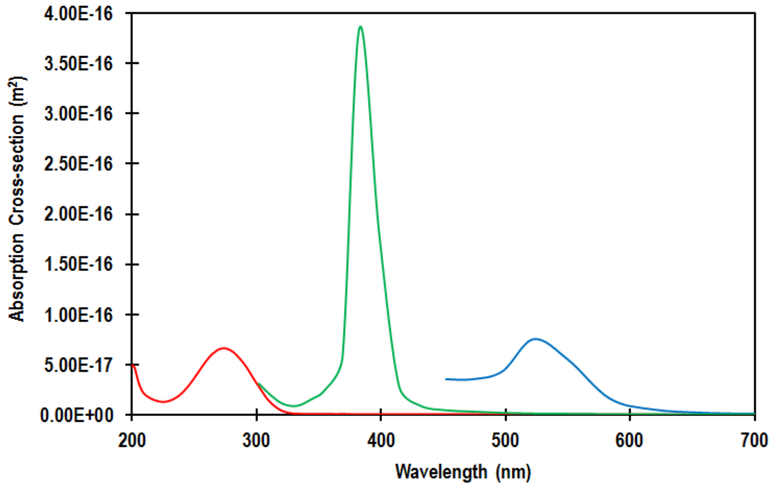

Figure S5.1. Absorption cross-section spectra of Co nanoparticles (red) in comparison with Au (blue) and Ag (green) nanoparticles.

Supporting References

1. Néel, L. Théorie du traînage magnétique des ferromagnétiques en grains fins avec application aux terres 5 cuites *Ann. Géophys.* **5**, 99-136 (1949).
2. Coffey, W. T.; Crothers, D. S. F.; Kalmykov, Yu. P.; Massawe, E. S.; Waldron, J. T. Exact analytic formula for the correlation time of a single-domain ferromagnetic particle. *Phys. Rev. E* **49**, 1869 (1994).
3. Gong, W.; Li, H.; Zhao, Z.; Chen, J. *J.* Ultrafine particles of Fe, Co, and Ni ferromagnetic metals. *Appl. Phys.* **69,** 5119 (1991).
4. Dormann, J. L.; D'Orazio, F.; Lucari, F.; Tronc, E.; Prene, P.; Jolivet, J. P.; Fiorani, D.; Cherkaoui, R.; Nogue's, M. Thermal variation of the relaxation time of the magnetic moment of g-Fe2O3 nanoparticles with interparticle interactions of various strengths. *Phys. Rev. B* **53**, 14291-297 (1996).
5. Mohenry, M. E.; Majetich, S. A.; Artman, J. O.; DeGraef, M.; Staley, S. W. Superparamagnetism in carbon-coated Co particles produced by the Kratschmer carbon arc process. *Phys. Rev. B* **49,** 11358 (1994).
6. Johnson, P. B.; Christy, R. W. Optical Constants of Transition Metals: Ti, V, Cr, Mn, Fe, Co, Ni, and Pd. *Phys. Rev.* **9**, 5056 (1974).
